# Saccade-vergence properties remain more stable over short-time repetition under overlap than under gap task: a preliminary study

**DOI:** 10.3389/fnhum.2014.00372

**Published:** 2014-06-02

**Authors:** Alexandre Lang, Chrystal Gaertner, Elham Ghassemi, Qing Yang, Christophe Orssaud, Zoï Kapoula

**Affiliations:** ^1^IRIS team Physiopathology of Binocular Motor Control and Vision, Centre National de la Recherche Scientifique – Université Paris DescartesParis, France; ^2^Service d’Ophtalmologie, Hôpital Européen Georges PompidouParis, France

**Keywords:** automatic, controlled, gap, overlap, rehabilitation

## Abstract

Under natural circumstances, saccade-vergence eye movements are among the most frequently occurring. This study examines the properties of such movements focusing on short-term repetition effects. Are such movements robust over time or are they subject to tiredness? 12 healthy adults performed convergent and divergent combined eye movements either in a gap task (i.e., 200 ms between the end of the fixation stimulus and the beginning of the target stimulus) or in an overlap task (i.e., the peripheral target begins 200 ms before the end of the fixation stimulus). Latencies were shorter in the gap task than in the overlap task for both saccade and vergence components. Repetition had no effect on latency, which is a novel result. In both tasks, saccades were initiated later and executed faster (mean and peak velocities) than the vergence component. The mean and peak velocities of both components decreased over trials in the gap task but remained constant in the overlap task. This result is also novel and has some clinical implications. Another novel result concerns the accuracy of the saccade component that was better in the gap than in the overlap task. The accuracy also decreased over trials in the gap task but remained constant in the overlap task. The major result of this study is that under a controlled mode of initiation (overlap task) properties of combined eye movements are more stable than under automatic triggering (gap task). These results are discussed in terms of saccade-vergence interactions, convergence-divergence specificities and repetition versus adaptation protocols.

## INTRODUCTION

Gaze redirection in three-dimensional space is most frequently based on combination of saccades and vergence. Vergence eye movements made in combination with saccades have been shown to be accelerated relative to isolated vergences, while saccades combined with vergence eye movements prove markedly slower when compared with isolated saccades (Ono et al., 1978; Kenyon et al., 1980; Enright, 1984; Erkelens et al., 1989; Maxwell and King, 1992; Zee et al., 1992; Collewijn et al., 1997; Van Leeuwen et al., 1998; Yang and Kapoula, 2004). Beyond the theoretical controversies surrounding the neurophysiological basis for the binocular control of eye movements (Kapoula et al., 2008; King, 2011), saccade-vergence interactions are of particular interest in the context of oculomotor rehabilitation. Vergence anomalies and more specifically convergence insufficiency is an increasingly common vision disorder promoted by visually demanding tasks undertaken at proximal distances relative to the subject (Von Noorden and Campos, 2002). The orthoptic rehabilitation of deficits and weaknesses in vergence usually consists in the repetition of isolated vergence along the median plane (Lavrich, 2010). This standard procedure has been shown to improve ocular motor performance by decreasing the latency (Bucci et al., 2004a) and duration (Jainta et al., 2011) of eye movements, on the one hand, and increasing the accuracy (Van Leeuwen et al., 1999; Bucci et al., 2004b) and velocity (Jainta et al., 2011) of eye movements, on the other.

Quantitative studies assessing the effects of repetitive eye movements on vergence are scarce. Moreover, in most of the studies conducted to date, eye movements are both predictable and triggered by the use of a haploscope (Yuan and Semmlow, 2000; Kim et al., 2011). Devices of this kind dissociate disparity and accommodation drivers. In a previous study, Jainta et al. (2011) triggered step vergence movements with targets located in the real space along the median plane while carefully avoiding any vergence-accommodation conflicts. The eye movement demands for this study consisted in convergence and divergence pseudo-randomly intermixed. Under most natural circumstances, isolated vergence movements are unusual and in light of the aforementioned studies it nevertheless remains unclear as to whether or not the repetition of combined eye movements can change the properties of vergence. Therefore, the current study investigates modifications in the spatial and temporal properties of combined eye movements due to repetition under ecological conditions in which targets are located in the surrounding milieu and the demands made on vergence are unpredictable.

**FIGURE 1:**
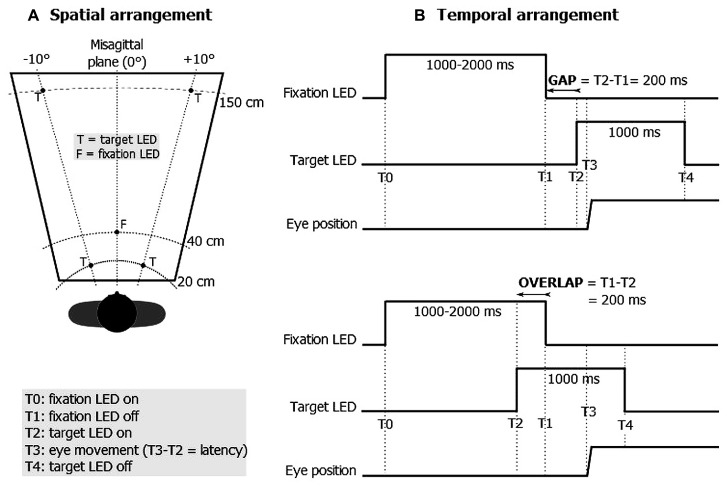
**Illustration of the spatial (A) and temporal (B) arrangement of the stimuli**.

Distinct modes of triggering eye movements have been proposed in the experimental literature based on the so-called “gap” and “overlap” paradigms (Saslow, 1967). The stimulation principle consists in displaying two visual signals successively: a fixation stimulus is first used in order to stabilize the gaze followed by a target stimulus which triggers eye movements. In the gap task, the fixation stimulus disappears briefly (e.g., 200 ms) before the target stimulus is displayed; in the overlap task, a short (e.g., 200 ms) overlapping of the fixation and the target stimulus is applied (see **Figure 1B**). Compared with the overlap task, the gap task was shown to reduce the latency of saccades (Saslow, 1967; Fischer, 1987; Goldring and Fischer, 1997) and, to a lesser extent, of both vergence (Tam and Ono, 1994) and combined eye movements (Takagi et al., 1995; Coubard et al., 2004; Bucci et al., 2005). This gap-overlap effect is highly robust spanning a fairly wide age range (Bucci et al., 2005; Yang et al., 2006; Yang and Kapoula, 2008).

One objective of the present study is to provide the basic principles required for developing novel rehabilitation procedures based on ecological eye movements which would be triggered under precise timing conditions. It is therefore of paramount importance that we identify the triggering mechanisms to be trained. Interestingly, automatic and controlled saccades, as triggered under gap and overlap tasks, have been found to involve specific adaptive mechanisms (Deubel, 1995; Alahyane et al., 2007; Pélisson et al., 2010; Gerardin et al., 2012). In particular, some adaptation studies point toward the generalizing effects obtained by repeating controlled saccades as opposed to automatic ones (Alahyane et al., 2007). It is likely that such effects can be generalized to the mere repetition of eye movements, specifically to combined saccade-vergence movements. There is clearly a need to investigate such aspects in laboratory conditions in order to provide knowledge of both theoretical and clinical significance. As a first step, this study is aimed at characterizing both the spatio-temporal properties and short-term repetition effects of combined eye movements performed during gap and overlap tasks in healthy young adults.

## MATERIALS AND METHODS

### ETHIC STATEMENT

The eye movement investigation adhered to the tenets of the Declaration of Helsinki and was approved by the local human experimentation committee (CPP Ile de France, No: 07035, Hospital Necker, Paris). Written consent was obtained from all subjects after the nature of the examination had been explained.

### SUBJECTS

Twelve healthy optometry students (22.8 ± 1.5 years) participated in the experiment. All of them had normal or corrected-to-normal vision. Binocular vision was assessed by the TNO (Test of Netherlands Organization; Richmond Products, Boca Raton, FL, USA); stereoscopic acuity was normal for each individual, i.e., 60 s of arc or smaller. The proximal point of convergence was 68 ± 15 mm for the group of subjects.

### STIMULI AND PROCEDURE

The stimuli consisted of five red light-emitting diodes (LEDs) of 2 mm in diameter which were positioned at eye level along three isovergence circles at 20, 40, and 150 cm in either perfect alignment with the midsagittal plane (0°) or 10° to the right or left of the midsagittal plane (see **Figure 1A**). Four LEDs were used as a target (T) and the LED located at the center (i.e., at 40 cm along the midsagittal plane) was used as a fixation (F) point. Each trial began by lighting the central fixation LED during a random period of 1000–2000 ms. One of the target LEDs was turned on for a period of 1000 ms at either 200 ms before (overlap task) or after (gap task) the central fixation LED was extinguished (see **Figure 1B**).

Subjects performed 80 convergent and 80 divergent movements combined with saccades. The number of rightward and leftward saccade components proved identical and the different combined movements were counterbalanced within subjects. Because we wanted to focus on repetition effects, subjects performed these movements in one task only: seven subjects were assigned to the overlap task, the others to the gap task. Each subject was seated in a chair equipped with a chinrest in order to stabilize their head in front of the visual display. The subjects were then instructed to fixate the LED as accurately as possible.

### EYE MOVEMENT RECORDING

Horizontal movements from both eyes were recorded simultaneously using the EyeLink II device (SR Research Ltd., Mississauga, ON, Canada). Each channel was sampled at 250 Hz. The system was calibrated with a spatial resolution of 0.025° in pupil-corneal reflection mode and a saccade event resolution of 0.05°.

### DATA SELECTION AND PARAMETER EXTRACTION

Raw data consisted of two signals representing horizontal positions of the left (LE) and the right (RE) eyes. These two raw signals were calibrated on the basis of a preliminary calibration task during which subjects performed four leftward and four rightward 10° saccades on a computer screen placed at 55 cm. From the calibrated signals we derived the conjugate signal by calculating the average position of both eyes, i.e., (LE + RE)/2. We also derived the disconjugate signal by calculating the differential position between both eyes, i.e., (LE-RE). After conducting low-pass filtering with the aid of a Gaussian finite impulse response filter (gain 0.1 at 85 Hz), the eye velocity of either the conjugate or disconjugate signal was computed using a symmetrical two-point differentiator.

The detection of either saccades or vergence was performed automatically using these calibrated conjugate, disconjugate and velocity signals. For the saccade component (conjugate signal), the beginning and end of the saccades were defined in terms of the precise moment in time when the eye velocity exceeded or dropped below 45°/s. This procedure concerned both the first, principal saccade of the combined movements as well as any observed subsequent corrective saccades. For the vergence component (disconjugate signal), the beginning and end of vergence movements were defined in terms of the precise moment in time when the eye velocity exceeded or dropped below 5°/s. These criteria are standard (Takagi et al., 1995; Yang et al., 2002). After this procedure, each movement was carefully scrutinized by an investigator.

### DATA ANALYSIS

Eye movements contaminated by blinks (1.9% of trials) and anticipatory eye movements with latencies in either the saccade or vergence component <80 ms (13% of trials) were rejected. Movements with latencies >400 ms (1.3% of trials) were also discarded. Indeed, this latency value is the upper limit in adults for slow saccades (Fisher et al., 1997). Finally, some movements were not taken into account either because one of the two components (saccade or vergence) did not change its position (1% of trials) or because the signal of at least one eye was partially lost during the movement (4.4% of trials). In total, 78.4% of the expected trials were analyzable.

The following sections deal with the latency, the mean and peak velocities and the accuracy in these movements. The mean velocity was defined as the ratio between the amplitude and the duration of each component. With respect to saccades, the mean velocity concerns only the first saccade as corrective saccades were not included in the velocity analysis. In so far as vergence is concerned, the mean velocity includes the whole movement from its initiation to the final steady state position. The accuracy was defined as the ratio between the measured amplitude and the target amplitude, for both the saccade (i.e., including corrective saccades) and the vergence components. The required convergence (8.5°) and divergence (6.3°) amplitudes were calculated in terms of a 60-mm mean interpupillar distance.

### STATISTICAL ANALYSIS

Data were analyzed using a linear-mixed effects model implemented in R-Development-Core-Team (2011; lmer from package lme4, Pinheiro and Bates, 2000; Venables and Smith, 2000), similarly to what was done in our previous study (see Jainta et al., 2011). In this study, we used the mixed effect method to analyze a range of oculomotor behaviors, namely: repetitive eye movements, component eye movements (saccade versus vergence), type of eye movement (convergent versus divergent). The linear-mixed effect model served also to test the interactions between those different categories of eye movement cited above.

Moreover, given that subjects were nested within the task (gap or overlap), they could not be considered as random factors in this model. The ANOVA tested whether the main effect of task and the interactions of task with component and with type of eye movements were significant. Other main effects (component, type of eye movements) and interaction between such factors were already tested in the linear-mixed effects model.

## RESULTS

### QUALITATIVE INSPECTION OF THE DATA

**FIGURE 2:**
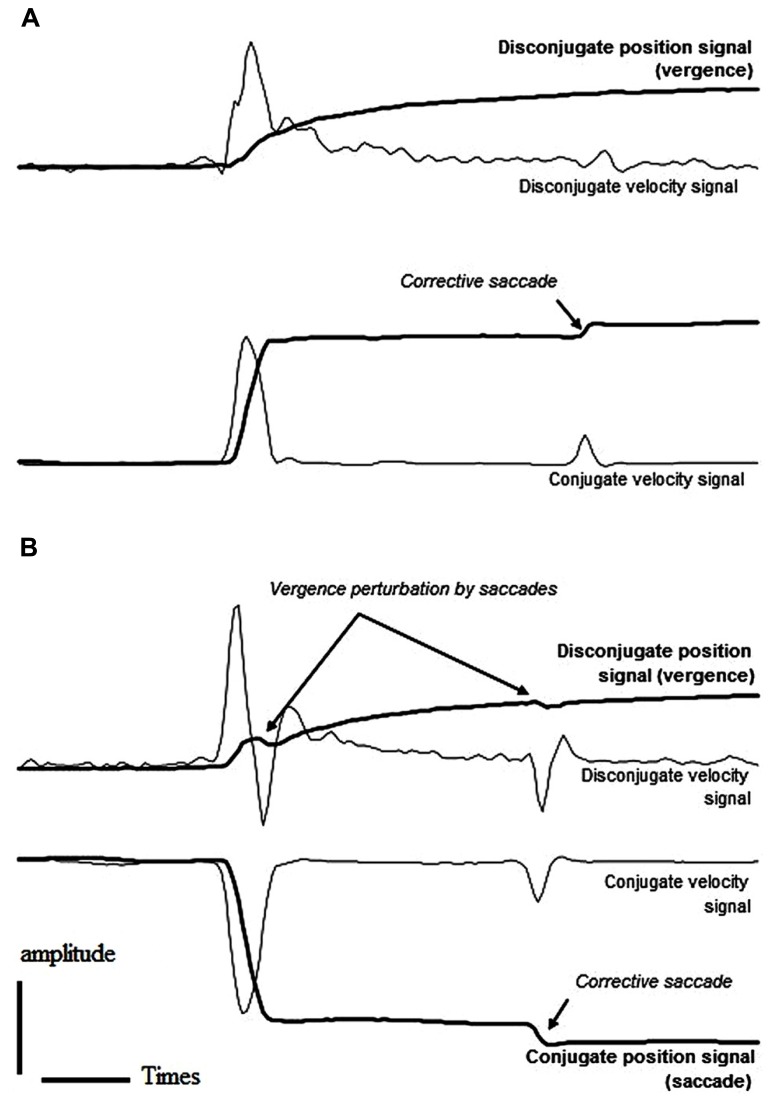
**Example saccade and vergence position and velocity signals from a subject performing right (A) and left (B) saccades combined with divergence in the gap task.** In both cases, the subject made a corrective saccade. The main and the corrective saccades did not perturb vergence smoothness in the first case **(A)** while they caused a local change in vergence direction in the second case **(B)** as indicated by the disconjugate velocity signal (see the text for more details).

As indicated in **Table 1**, a large percentage of the anticipatory movements occurred in the gap task for both divergent (29%) and convergent (15%) movements; anticipatory movements in the overlap task were comparatively few in number making up only 3 and 5% of the convergent and divergent movements, respectively. This result is in keeping with the study of Takagi et al. (1995) in which the authors reported a particularly high rate of anticipations for the divergence component of combined movements. In addition, more than half of the remaining non-anticipatory movements presented at least one corrective saccade; this was the case for either task (gap/overlap) and for either type of movement (convergent/divergent). Interestingly, corrective and/or main saccades perturbed the vergence smoothness in almost half of the divergent movements while smoothness of vergence was preserved in the large majority of convergent movements, in either gap or overlap tasks. **Figure 2** shows typical saccade (conjugate) and vergence (disconjugate) signals from the same subject performing divergent combined movements in the gap task. In the first case (**Figure 2A**), the absolute vergence signal remains relatively smooth while in the second case (**Figure 2B**), both the principal and the corrective saccades perturb the absolute vergence component. We considered that the absolute vergence smoothness was perturbed when the signal indicated an abrupt step-like change (see traces in **Figure 2**).

**Table 1:** Percentage of rejected anticipatory movements (ANT) and of retained movements containing corrective saccades (CS) or in which the vergence component was perturbated by saccades (VP) listed per task (gap and overlap) and type of movement (CONV, convergent movements; DIV, divergent movements).

	GAP		OVERLAP	
	CONV	DIV	CONV	DIV
ANT	15	29	3	5
CS	51	54	60	71
VP	6	48	12	40

Mean values for each parameter, each component (saccade, vergence), each type of movement (convergent, divergent) and each task (gap, overlap) are reported in **Table 2**.

**Table 2:** Mean values (with standard errors) listed per component (convergence, saccade-convergence, divergence and saccade-divergence) and task (gap and overlap) for each parameter (LAT, latency; ACC, accuracy; MVE, mean velocity; PVE, peak velocity).

Combined convergent movements				
Parameter	Convergence		Saccade-convergence	
	Gap	Overlap	Gap	Overlap
LAT (ms)	149 (2.25)	160 (2.05)	184 (2.01)	207 (2.49)
ACC	0.80 (0.02)	0.76 (0.01)	0.76 (0.01)	0.72 (0.01)
MVE (deg/s)	19 (0.60)	19 (0.44)	152 (2.42)	151 (1.67)
PVE (deg/s)	139 (3.70)	141 (2.12)	277 (4.32)	274 (2.80)

**Combined divergent movements**				
**Parameter**	**Divergence**		**Saccade-divergence**	
	**Gap**	**Overlap**	**Gap**	**Overlap**

LAT (ms)	166 (3.03)	183 (2.28)	194 (3.00)	223 (2.79)
ACC	0.69 (0.02)	0.79 (0.01)	1.00 (0.01)	0.96 (0.01)
MVE (deg/s)	10 (0.34)	10 (0.23)	182 (2.58)	183 (1.41)
PVE (deg/s)	75 (2.73)	78 (1.37)	309 (4.36)	309 (2.20)

### PREPARATION OF COMBINED EYE MOVEMENTS IS INFLUENCED BY THE TASK BUT NOT BY REPETITION

Firstly, as illustrated in **Table 2**, the latencies were longer in the overlap task than in the gap task no matter the component (saccade/vergence) and type of movement (convergent/divergent; *F*_1,3056_ = 115.97, *p* < 0.0001). A significant interaction between the task and component (*F*_1,3056_ = 10.36, *p* < 0.001) can be attributed to a stronger task effect for the saccade (*F*_1,1530_ = 83.93, *p* < 0.0001) than for the vergence component (*F*_1,1530_ = 31.91, *p* < 0.0001).

Secondly, latencies were longer for the saccade as compared to the vergence component in both gap (*t* = -4.50, *p* < 0.0001) and overlap (*t* = -4.64, *p* < 0.0001) tasks. These differences occurred for both convergent and divergent combined movements (*t* = 1.84, *p* = 0.07 in gap; *t* = 1.07, *p* = 0.27 in overlap).

Lastly, repetition of the combined movements did not modify their latencies in either the gap (*t* = -1.17, *p* = 0.25) or overlap (*t* = -1.39, *p* = 0.16) tasks. The absence of a significant interaction between trial and component (*t* = 1.85, *p* = 0.06 in gap; *t* = 0.60, *p* = 0.55 in overlap) or between trial and type of movement (*t* = 0.85, *p* = 0.40 in gap; *t* = 0.84, *p* = 0.40 in overlap) indicates that repetition had no effect on either type of combined eye movements tested in gap and overlap tasks.

### THE VELOCITY OF COMBINED EYE MOVEMENTS IS INFLUENCED BY REPETITION IN GAP BUT NOT IN OVERLAP TASKS

In terms of both mean and peak velocity, combined eye movements were similar in gap and in overlap tasks as indicated by the non-significant effects of the task factor (mean velocity: *F*_1,3056_ = 0.03, *p* = 0.86; peak velocity: *F*_1,3056_ = 0.16, *p* = 0.69).

Velocities were significantly higher for the saccade as compared to the vergence components (mean velocity: *t* = -14.19, *p* < 0.0001 in gap; *t* = -14.1, *p* < 0.0001 in overlap – peak velocity: *t* = -2.68, *p* < 0.007 in gap; *t* = -2.53, *p* < 0.013 in overlap). This was the case for both types of movements (combined convergent and combined divergent) and for both tasks (gap/overlap). As shown in **Table 2**, the convergence component in the gap task has smaller mean and peak velocity compared to saccade component. It is also the case for the overlap task and for the both types of combined movements; divergent and convergent (i.e., their vergence components have always lower velocities than their saccade component, all *p* < 0.05).

Concerning the repetition, the mean and peak velocities decreased over the course of the trials in the gap task (mean velocity: *t* = -6.76, *p* < 0.0001; peak velocity: *t* = -3.45, *p* < 0.0004) and remained constant in the overlap task (mean velocity: *t* = 0.23, *p* = 0.81; peak velocity: *t* = 0.22, *p* = 0.8252). In the overlap task, non-significant interaction between trial and component (mean velocity: *t* = -0.2, *p* = 0.83; peak velocity: *t* = -0.36, *p* = 0.71) and trial and type of movement (mean velocity: *t* = -1.03, *p* = 0.29; peak velocity: *t* = -0.4, *p* = 0.6836) indicate that velocities remained constant over the trials in this task. In the gap task, the interaction between trial and component was significant relative to the mean velocity (*p* < 0.0001) and non-significant relative to the peak velocity (*p* = 0.54). In contrast, the interaction between trial and type of movement was significant for both mean and peak velocities (*p*s < 0.0001 and 0.025, respectively). Notwithstanding the aforementioned interactions, the repetition effect was significant in any gap task.

### THE FINAL ACCURACY IN COMBINED EYE MOVEMENTS IS INFLUENCED BY THE TASK AND BY REPETITION IN GAP TASK

The main effect of task (gap, overlap) is not significant for the accuracy (*F*_1,3036_ = 0.06, *p* = 0.81) but there is a significant interaction between the task and component (*F*_1,3036_ = 11.8, *p* < 0.001). Indeed, the accuracy was significantly better in the gap than in the overlap task (*F*_1,1510_ = 7.67, *p* < 0.006) for the saccade component while it did not differ significantly (*F*_1,1530_ = 3.16, *p* = 0.08) for the vergence component (see **Table 2**).

The interaction between the component and type of movement is significant in both tasks, gap (*t* = -8.88, *p* < 0.0001) and overlap (*t* = -7.91, *p* < 0.0001). On the one hand, the saccade component presents a better accuracy in divergent as compared to convergent movements in both tasks (gap: *t* = 7.78, *p* < 0.0001; overlap: *t* = 17.95, *p* < 0.0001). On the other hand, the vergence component presents a lower accuracy in divergent as compared to convergent movements in the gap task (*t* = -5.75, *p* < 0.0001) whereas in the overlap task, the type of movement does not differ significantly (*t* = 0.87, *p* = 0.38). Such findings indicate reciprocal interactions between saccade and vergence which differ according to the type of movement.

As was the case for the velocities, the accuracy decreased over trials in the gap task (*t* = -6.32, *p* < 0.0001) and remained constant in the overlap task (*t* = 0.63, *p* = 0.53). In the overlap task, non-significant interaction between trial and component (*t* = -0.8, *p* = 0.44) or between trial and type of movement (*t* = -0.71, *p* = 0.49) indicate that the accuracy remained constant over trials in this task. In the gap task, the interaction between the trial and component is significant (*t* = -2.68, *p* < 0.006) while interaction between the trial and type of movement is not significant (*t* = -1.08, *p* = 0.26). The decrease of the accuracy is significant for the saccade component (*t* = -3.52, *p* < 0.001) as well as for the vergence component (*t* = -5.06, *p* < 0.0001) in the gap task.

A summary of the results are shown in **Table 3** for the effect of task, component and type of movement. Latencies are shorter in the gap tasks, velocities are higher for the saccade component; the accuracy of saccade component is better when combined with divergence than with convergence while the vergence component is more accurate for convergence than divergence movement in the gap task.

**Table 3:** Summary of the statistically significant results (in bold) for the effect of task (gap/overlap), component (saccade/vergence), and type of movement (convergent/divergent) for each parameter (latency, mean velocity, peak velocity, and accuracy).

	Task	Component	Type of movement
	(Gap/overlap)	(Saccade/vergence)	(Convergence/divergence)
Latency	**Gap < overlap**	**Saccade > vergence**	NS
			
Mean velocity	NS	**Saccade > vergence**	NS
Peak velocity	NS	**Saccade > vergence**	NS
Accuracy	NS	**For saccade gap > overlap**	**Saccade component of divergent movement is more accurate than the saccade component of convergent movement; for gap only, the convergence component is more accurate than the divergence component**

*NS, non-significant.*

**Table 4** summarizes significant repetition effect. In the gap condition only, mean velocity, peak velocity, and accuracy decrease with repetition of movement.

**Table 4:** Summary of the statistically significant results (in bold) for the repetition effect of task (gap/overlap).

	Gap	Overlap
Latency	NS	NS
		
Mean velocity	**Decrease**	NS
		
Peak velocity	**Decrease**	NS
		
Accuracy	**Decrease**	NS
		

*NS, non-significant.*

## DISCUSSION

In this study, we examined the spatio-temporal properties of repeated saccades combined with convergence and divergence movements in gap and overlap tasks. The main results are the following: (1) The gap task led to faster initiation than the overlap task for both saccade and vergence components as well as to a better final accuracy in the saccade component (i.e., including corrective saccades). (2) In both tasks, saccades were initiated later and executed faster than their combined vergence; divergent saccades had a better accuracy than the convergent saccades while for the vergence component, convergence had a better accuracy than the divergence. (3) Latency in gap and overlap tasks as well as accuracy and mean and peak velocities in the overlap tasks remained constant over trials; accuracy and mean and peak velocities decreased over trials in the gap task. These results will be discussed below.

### GAP-OVERLAP EFFECT FOR BOTH COMPONENTS OF COMBINED MOVEMENTS

Both the saccade and the vergence components of the combined movements recorded in this study were initiated faster during the gap task than during the overlap task, presenting this *gap effect*. Such effect has been extensively investigated for saccades, and called the *gap effect* (Fischer, 1987; Tam and Ono, 1994; Takagi et al., 1995; Goldring and Fischer, 1997; Coubard et al., 2004; Bucci et al., 2005). Shorter latencies in the gap task have been attributed to the disengagement of either attention (Fischer et al., 1993; Tam and Stelmach, 1993) or fixation (Kingstone and Klein, 1993) during the gap period thus facilitating the visual shift to the target. Moreover, overlapping the target with the fixation stimulus would most likely increase the decision-to-move requirement. Several lines of neurophysiological evidence indicate that frontal eye fields (FEF) are specifically involved in the preparation of voluntary eye movements while automatic eye movements recruit shorter cerebral circuits which may or may not include the posterior cortex (Hanes and Schall, 1996; Schiller and Tehovnik, 2005). However, attentional and decision-making processes associated with the preparation of eye movements are also thought to involve different neural pathways mediated by the parietal cortices and the FEF, respectively (Fischer and Weber, 1993). We believe that eye movements triggered in the overlap task involve more voluntary or controlled processes than in the gap task. Indeed, transcranial magnetic stimulation (TMS) of the right posterior parietal cortex (PPC) causes an increase in the latency variability for saccades in the gap task while decreasing variability in the overlap task (Kapoula et al., 2011).

The accuracy in the vergence component, the mean and peak velocities obtained for both components, were all not significantly different between the gap and overlap task. One of this study’s more outstanding observations concerned the final accuracy of the saccade component (including corrective saccades) which was better in the gap than in the overlap task. We have no formal explanation for this result but one might speculate that the computation of the saccade metric is perhaps less influenced by parallel processing in the vergence component. If the combined movement in the overlap task is triggered by the frontal area, given that vergence and saccade retinal maps sub-areas are proximal within the FEF (Gamlin and Yoon, 2000; Yang and Kapoula, 2011), stronger reciprocal interaction between saccade and vergence metrics information might occur; while for the gap task, metric information could be provided by the parietal cortex for the saccade and by frontal are for the vergence that are more distant and thus less interacting. Speculative hypotheses aside, these findings are clearly in need of further investigation.

### SPECIFIC RECIPROCAL SACCADE-VERGENCE INTERACTIONS FOR CONVERGENT AND DIVERGENT COMBINED MOVEMENTS

In both tasks, the vergence component was executed both before and more slowly than the saccade component. And indeed, this observation is compatible with earlier findings (Coubard et al., 2004). Interestingly, reciprocal interactions between the component (saccade, vergence) and the type of movement (convergent, divergent) were found in terms of accuracy: divergent saccades had a better accuracy than the convergent saccades while for the vergence component, convergence had a better accuracy than the divergence. Many studies have provided substantial evidence for vergence and saccade synergies by demonstrating that the peak velocity of the vergence component of combined movements is higher than that of isolated vergence, while the peak velocity of the saccade component is lower than that of isolated saccade (Ono et al., 1978; Kenyon et al., 1980; Enright, 1984; Erkelens et al., 1989; Maxwell and King, 1992; Zee et al., 1992; Collewijn et al., 1997). In other words, the observed reciprocal interactions are characterized by an acceleration of vergence triggered by the saccade and by a deceleration of the saccade triggered by vergence. The results of this study suggest that such reciprocal interactions affect the accuracy in both components differentially when the combined movements are either convergent or divergent: our data (see **Table 2**) indicate a greater accuracy in the divergent saccade component as compared to the convergent saccade component, whereas the accuracy in divergence, at least with respect to the gap task, is worse than the accuracy in convergence. In other words, it would appear as if the convergence component influences the combined saccade to a greater extent than does the divergence component influence the corresponding saccade component.

Differential processing for convergence and divergence has already been suggested. For instance, Mays (1984) identified distinct convergence and divergence cells in the mesencephalic reticular formation which presented similar activation-characteristics: convergence cell activity increased while divergence cell activity decreased for increased convergence. The anatomical separation of discharging convergence and divergence neurons was also demonstrated within the deep cerebellar nuclei, the nucleus reticularis tegmenti pontis and the preacurate region (Gamlin, 2002). Distinct control of divergence was also demonstrated at the cortical level, specifically in the prearcuate cortex rostral to the anterior bank of the arcuate sulcus in which neurons have been found to display activity during either convergent or divergent movements (Gamlin and Yoon, 2000). Evidence for distinct divergence and convergence systems also exists in healthy humans. Using electroencephalography, Tzelepi et al. (2004) recorded higher activation in the posterior and central cortex for convergence while activation related to divergence was mainly distributed ventrally from the occipital cortex. So the results presented here might reveal a trade-off between saccade and vergence component accuracy, the vergence being privileged for convergence movements and the saccade privileged for divergent movement. This result is relevant in real-life situations.

### REPETITION DECREASES THE ACCURACY AND THE VELOCITIES OF COMBINED MOVEMENTS IN GAP BUT NOT IN OVERLAP TASKS

To the best of our knowledge, gap and overlap differences induced by repetition have never been examined in eye movements triggered by simple-step targets. And yet, this issue has been indirectly addressed in adaptation studies in which a double-step target paradigm is used to trigger saccades (McLaughlin, 1967). Contrary to the standard, simple-step stimulation, this protocol consists of changing the location of the visual target during the primary saccade execution thus inducing a post-saccadic error. Numerous studies have demonstrated that repeated iterations of saccades in such a paradigm lead to the progressive adjustment of the saccade amplitude, suggesting the existence of adaptive mechanisms (Takagi et al., 1995; Hopp and Fuchs, 2004).

The protocol used in the current study presents some important differences compared with the aforementioned adaptation studies, especially in terms of visual stimulation (simple-step vs. double-step target), eye movements (saccades combined with vergence vs. saccades only) and mode of triggering (automatic/controlled vs. automatic/voluntary; but see also Introduction). However, both protocols deal with repetition of eye movements. Our results indicate progressive changes over trials in the properties of saccades combined with vergences which are specific to their mode of initiation. In particular, the accuracy and the mean and peak velocities of both components of combined eye movements decreased in the gap task and remained constant in the overlap task. Because subjects were required to accurately follow the visual target in both this study’s repetition task as well as in previous adaptation tasks, it could be argued that controlled or voluntary triggering are more advantageous than automatic initiation in terms of the immediate performance benefit due to training. Indeed, functional links between saccadic adaptation and attention control has already been hypothesized based on anatomical overlap between adaptation substrates for automatic/voluntary saccades and dorsal/ventral specialization for intentional/stimulus-driven shifts of attention (Gerardin et al., 2012). Pending future investigations, we propose that similar links exists concerning combined eye movements in simple-step paradigms. The preservation of accuracy in the overlap task as compared with the observed decrement in accuracy in the gap task could therefore be similarly attributed to the attention processing of the related visual inputs in so far as attention is thought to be maintained during overlap tasks and relaxed during gap tasks (Fischer et al., 1993; Tam and Stelmach, 1993).

### CLINICAL PERSPECTIVES

As mentioned in the introduction, clinical orthoptic rehabilitation of vergence insufficiencies typically involves the push-up technique in which the patient has to maintain fusion while fixating the tip of a pencil that is moved along the median plane toward the nose (Lavrich, 2010). One of the goals of this study was to formulate a basic account of the properties of combined saccade-vergence eye movements for use in innovative and potentially rehabilitative procedures. These rehabilitative procedures would themselves be based on ecological eye movements and the well-controlled timing of their activation. In light of these more pragmatic aims, our findings suggest that the simple repetition of combined eye movements, when initiated under a controlled mode (overlap task), would be better suited to the task of sustaining a less variable and more constant immediate ocular motor performance. Further research is needed in order to identify the impact, subsequent to such training, of associated learning effects on patients.

## Conflict of Interest Statement

The authors declare that the research was conducted in the absence of any commercial or financial relationships that could be construed as a potential conflict of interest.
